# Relationship of mottling score, skin microcirculatory perfusion indices and biomarkers of endothelial dysfunction in patients with septic shock: an observational study

**DOI:** 10.1186/s13054-019-2589-0

**Published:** 2019-09-11

**Authors:** Sigita Kazune, Anastasija Caica, Karina Volceka, Olegs Suba, Uldis Rubins, Andris Grabovskis

**Affiliations:** 1 Department of Anesthesiology, Hospital of Traumatology and Orthopedics, 22 Duntes Street, Riga, LV-1013 Latvia; 2 Laboratory of Biophotonics, Institute of Atomic Physics and Spectroscopy, University of Latvia, 3 Jelgavas Street, Riga, LV-1004 Latvia; 3 Department of Human and Animal Physiology, Faculty of Biology, University of Latvia, 1 Jelgavas Street, Riga, LV-1004 Latvia; 4 Clinic of Toxicology and Sepsis, Riga East University Hospital, 2 Hipokrata Street, Riga, LV-1038 Latvia

**Keywords:** Sepsis, Tissue oxygenation, Microcirculation, Hyperspectral imaging

## Abstract

**Background:**

In patients with septic shock, the skin is often chosen for the evaluation of peripheral perfusion and oxygenation. Changes in skin microcirculatory vessel oxygen saturation and relative hemoglobin concentration can be described using a mottling score or captured with hyperspectral imaging. However, the effectiveness of the mottling score in assessing microcirculation remains to be shown. We hypothesize that the mottling score in patients with septic shock is related to skin microcirculatory perfusion indices quantified by hyperspectral imaging, biomarkers that reflect endothelium activation and damage, and clinical outcome.

**Methods:**

Hyperspectral imaging of the knee area was performed in 95 intensive care patients with septic shock enrolled in a single-center observational study to obtain relative oxy/deoxyhemoglobin concentration values and construct anatomical maps of skin microcirculatory saturation. The blood was sampled to obtain concentrations of thrombomodulin, plasminogen activator inhibitor-1 (PAI-1), soluble intercellular adhesion molecule-1 (ICAM-1), soluble vascular cell adhesion molecule-1 (VCAM-1), angiopoietin-2, and syndecan-1. The spectrophotometrically obtained skin microvascular perfusion indices were compared to the mottling score and biomarker concentration. The association between mottling score, skin microcirculatory perfusion indices, and 28-day mortality was also analyzed.

**Results:**

Microcirculatory oxygen saturation was significantly lower and total hemoglobin concentration was significantly higher in patients with a mottling score of 2 compared to those with a score of 0 (*p* = 0.02), with no difference between other scores. We found an association between microcirculatory oxygen saturation and PAI-1 levels (rho = − 0.3; *p* = 0.007). Increased mottling score and decreased microcirculatory oxygen saturation were predictive of 28-day mortality (mottling score 2 vs 0: OR 15.31, 95% CI 4.12–68.11; microcirculatory oxygen saturation: OR 0.90, 95% CI 0.85–0.95). Endothelial biomarkers did not increase the predictive value of skin microcirculatory perfusion indices.

**Conclusions:**

Higher mottling scores are associated with lower microcirculatory oxygen saturation but with significant overlap between scores. Microcirculatory oxygen saturation is a quantitative measure of peripheral oxygenation and is more specific than the mottling score in predicting 28-day mortality.

**Electronic supplementary material:**

The online version of this article (10.1186/s13054-019-2589-0) contains supplementary material, which is available to authorized users.

## Background

Suboptimal tissue oxygenation, a consequence of microcirculatory perfusion abnormalities, is thought to be an important step in the pathogenesis of septic shock [1]. Clinically, the skin is often chosen for the evaluation of peripheral perfusion and oxygenation. Decreased oxygen saturation and stagnation of red blood cells in the skin microvasculature can be seen as mottling. Mottling score (MS) has been developed for its semiquantitative assessment [2]. Changes in the amount and wavelength pattern of the light reflected from the skin can also be captured using hyperspectral imaging (HSI) which allows the quantification of skin microcirculatory indices, such as total hemoglobin concentration and oxygen saturation. The mottled areas of the skin have been shown to have reduced blood flow and low muscle O_2_ saturation [3, 4]. However, the effects of skin total hemoglobin concentration and oxygen saturation values on the clinical appearance of mottling are not well understood.

Understanding the underlying pathophysiologic mechanisms involved in mottling is important if the effectiveness of mottling for microcirculatory assessment is to be shown. Bourcier and colleagues found endothelial dysfunction in the mottled skin using vascular reactivity tests [5]. Other proposed mechanisms are the activation of the coagulation cascade and vasoconstriction due to high sympathetic tone [6, 7]. The measurement of circulating biomarkers of endothelial dysfunction in relation to skin microcirculatory indices could offer new data regarding the link between changes in cell adhesion, coagulation cascade, and glycocalyx shedding and skin dysoxia.

We hypothesize that in septic shock, the extent of mottling in the knee area (1) is related to skin microcirculatory perfusion indices obtained with hyperspectral imaging and (2) is associated with the concentration of circulating biomarkers of endothelial cell dysfunction, and (3) biomarkers and skin microcirculatory perfusion indices increase the predictive value of mottling score on mortality.

## Methods

The patients for this prospective observational study were recruited from a 16-bed mixed intensive care unit between March 2017 and December 2018. Consecutive adult (more than 18 years of age) patients with sepsis admitted to the unit were screened within 24 h of admission. Patients with septic shock were eligible for inclusion. Septic shock was defined as an organ dysfunction related to an infection [8] and the presence of hypotension uncorrected by fluid resuscitation. This group was chosen because of the high incidence of visible mottling reported in previous studies [9]. Patients with extensive wounds or skin discoloration in the knee and thigh areas unrelated to mottling were excluded.

The study protocol was approved by the Institutional Research Ethics Committee (26/23.02.2017), and all participants or their closest relatives provided written informed consent.

### Clinical management of patients

All clinical management was determined by local protocols. Clinical staff not involved in the study tailored therapy with fluids, vasopressors, and inotropes individually to maintain a mean arterial pressure of > 65 mmHg.

### Study design

Visual evaluation and hyperspectral imaging of the skin around the knee area were performed when a mean arterial pressure of > 65 mmHg was achieved, and there had been no change in vasopressor requirements for at least 1 h.

The following clinical information was collected from the patients’ hospital records: demographic data (age and sex), primary site of infection, and clinical and laboratory data necessary to calculate Acute Physiology and Chronic Health Evaluation (APACHE) II [10] and Sequential Organ Failure Assessment (SOFA) [11] scores. Survival status was recorded at 28 days.

Mottling of the anterior aspect of the knee was assessed visually on both legs. Patients were placed supine with the legs straight and at the level of the heart. Mottling score (MS) which describes the extent of the mottled area on the knee and thigh was determined on a 6-point scale ranging from 0 to 5 as described previously [2]. If mottling was present, then the leg with more prominent mottling was chosen for scoring and imaging. Mean arterial pressure, doses of vasopressor agents, partial pressure of oxygen and lactate in the arterial blood, and core temperature at the time of imaging were also recorded.

### Hyperspectral imaging equipment and measurement procedure

Hyperspectral images of the skin overlying the patella were acquired by a custom-built system consisting of multispectral camera Nuance EX (PerkinElmer, Alameda, USA) combined with a light source, both fixed on a tripod. An HSI data cube acquired from each patient contained 75 12-bit 1392 × 1024 pixel monochromatic images captured in the range of 450–820 nm with a step of 5 nm. All HSI cubes were saved as a set of lossless monochrome .tiff files. The processing of the HSI data was performed offline in semiautomatic mode using custom MATLAB (MathWorks, Natick, USA) code. The HSI image cube was divided into three clusters depending on the total hemoglobin concentration, obtaining precise skin regions with the highest pooling of blood. The oxygen saturation value at each pixel was calculated utilizing a three-layer optical model of the skin [12, 13] which infers the content of oxyhemoglobin, deoxyhemoglobin, and melanin from measured reflectance spectra. A full description of the image acquisition and analysis is provided in Additional file 1.

**Fig. 1:**
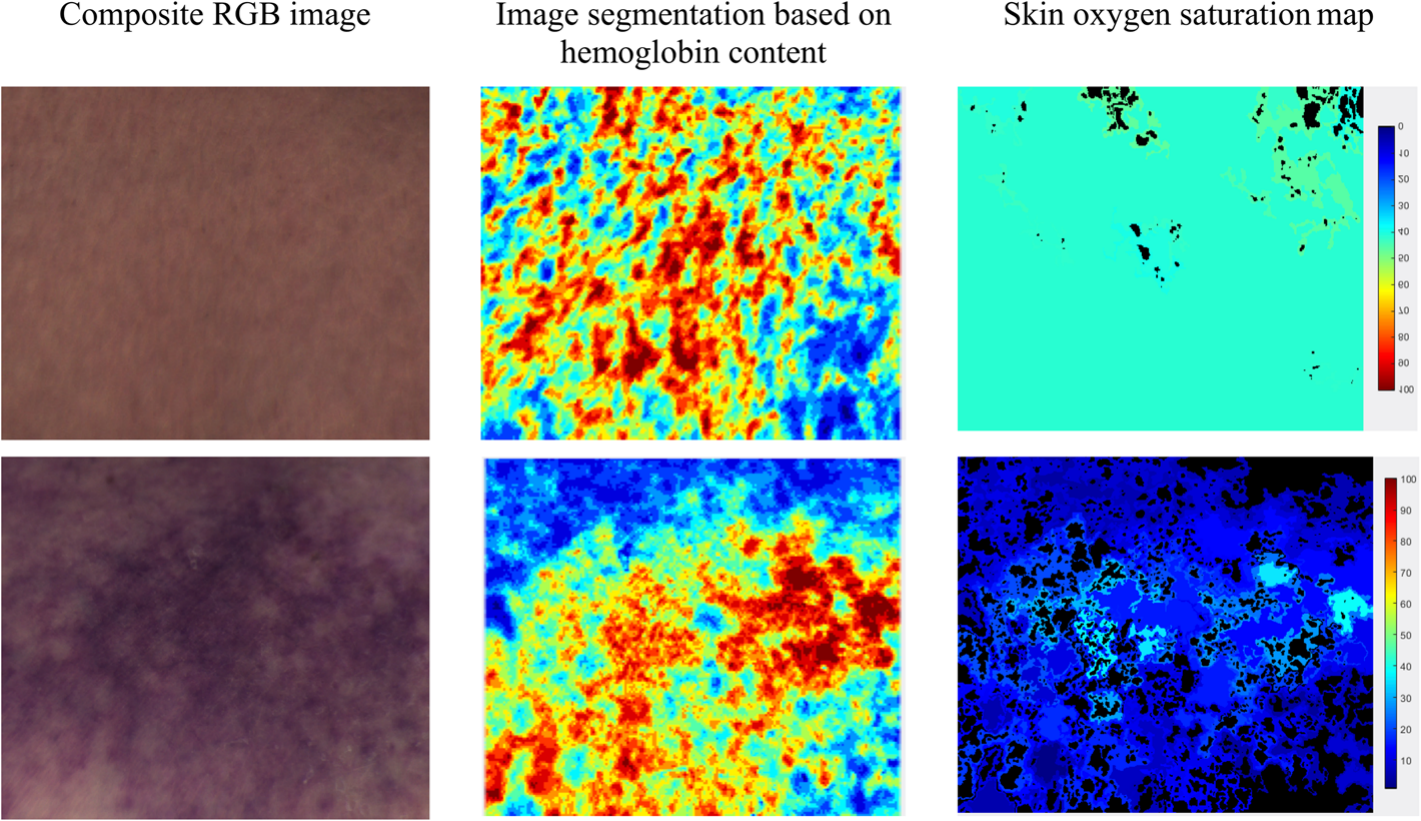
Images of two septic shock patients at different stages of processing. Segmented images were generated by dividing the hyperspectral cube into clusters with similar spectral properties that correspond to the degree of blood pooling. Skin oxygen saturation maps were generated by calculating μHbSO_2_ for each pixel in the region imaged. Each pixel was colored according to its value. Homogenous skin oxygenation is observed in a patient with higher μHbSO_2_. In the case of low μHbSO_2_, significant heterogeneity is present

The mean values of microcirculatory blood oxygen saturation percentage (μHbSO_2_) and relative total hemoglobin concentration (μHbtot) in arbitrary units (a.u.) were calculated from the region of the imaged skin with the highest pooling of blood. The obtained values for each patient were used for further statistical analysis. Examples of the images obtained at different stages of HSI processing are shown in Fig. 1.

### Biomarker analysis

Venous blood samples were collected from all patients in ethylene diamine tetraacetic acid tubes within 24 h of intensive care unit admission at the time of HSI. Within 30 min, the samples were centrifuged for 15 min at 1000 rpm, immediately aliquoted, frozen, and stored at − 80 °C until use. Plasma intracellular adhesion molecule-1 (ICAM-1), vascular cell adhesion molecule-1 (VCAM-1), and tissue plasminogen activator inhibitor-1 (PAI-1) were assayed by xMAP technology on a Luminex 200 analyzer (Luminex Corporation, Austin, USA) using a Milliplex MAP Human Sepsis Magnetic Bead Panel 1 kit (HSP1MAG-63K; Merck KGaA, Darmstadt, Germany). Plasma thrombomodulin was detected by the ELISA method using a human thrombomodulin/BDCA-3 Quantikine ELISA Kit (DTHBD0; R&D Systems, Inc., Minneapolis, USA). Plasma angiopoietin-2 was detected by ELISA using a human angiopoietin-2 Quantikine ELISA Kit (DANG20; R&D Systems, Inc., Minneapolis, USA). Plasma syndecan-1 was detected by ELISA using a human syndecan-1 ELISA kit (AB46506; Abcam plc., Cambridge, UK) and an Infinite®M200 analyzer (Tecan Trading AG, Switzerland). The intra-assay coefficient of variation was calculated after the analysis of two samples of low immunological marker concentration and two high concentration five times in a single assay run. The intra-assay coefficient of variation was 2.6%, which was in accordance with the manufacturer’s instructions.

### Statistical methods

All data in this study were analyzed using R version 3.3.2 (The R Foundation for Statistical Computing, GNU General Public License, Boston, USA) with Hmisc, rms, ROCR, and ggpubr packages. Demographic, hemodynamic, microcirculatory, and biomarker data were expressed as median (interquartile range) or counts (percentages). Statistical comparisons between survivors and non-survivors were performed by the Wilcoxon-Mann-Whitney two-sample rank-sum test. Data regarding skin μHbSO_2_ and μHbtot across patients with different MS were compared using the Kruskal-Wallis test with post hoc Mann-Whitney analysis. The association between endothelial biomarkers and MS or μHbSO_2_ was assessed using Spearman’s rank correlation analysis. Binary logistic regression was used to estimate the odds ratios (OR) and 95% confidence intervals (CI) for the association between 28-day survival and MS or μHbSO_2_. Discrimination in univariate models was assessed by the *C* statistic. We used multivariate logistic regression to model 28-day survival as a function of MS, μHbSO_2_, μHbtot, and concentrations of thrombomodulin, angiopoietin-2, ICAM-1, VCAM-1, PAI-1, and syndecan-1. A *p* value of less than 0.05 was considered statistically significant.

## Results

Ninety-five patients were recruited into the study. Hyperspectral images of 6 patients could not be used due to technical faults during acquisition, and these patients were excluded from the analysis. Thus, μHbSO_2_ and μHbtot obtained from HSI, clinical, and biomarker data were available for 89 patients, and their demographic and clinical data are included in Table 1.

**Table 1:** Patient demographic, hemodynamic, acid-base, and perfusion characteristics

	All patients, *n* = 89	28-day survivors, *n* = 64	28-day non-survivors, *n* = 25	*p* value
Demographics				
Age (years)	70 (62–78)	69 (61–78)	72 (58–77)	0.31
Male, *n* (%)		39 (61%)	14 (56%)	0.81
APACHE II	23 (18–27)	21 (17–25)	29 (23–33)	< 0.001
SOFA	9 (7–12)	8 (6–10)	12 (10–13)	< 0.001
Mechanical ventilation	32 (36%)	17 (43%)	15 (75%)	0.03
Source				
Respiratory	2 (26%)	11 (17%)	12 (48%)	n/a
Abdominal	31 (35%)	26 (41%)	5 (20%)	n/a
Urinary	26 (29%)	22 (34%)	4 (16%)	n/a
Others	9 (%)	5 (8%)	4 (16%)	n/a
Temperature (°C)	37.3 (36.7–38.5)	37.2 (36.8–38.4)	37.4 (36.6–38.9)	0.91
Hematocrit (%)	35 (29–39)	33 (28–39)	37 (32–39)	0.23
Hemoglobin (g/dL)	12.3 (10.4–13.8)	12.2 (10.4–13.8)	12.4 (10.9–13.9)	0.87
Bilirubin (mcmol/L)	17.5 (9.0–29.0)	18 (10–32)	15 (6–26)	0.25
Hemodynamics				
HR, beats/min	100 (91–115)	96 (91–103)	102 (94–112)	0.81
MAP, mmHg	75 (67–85)	79 (68–85)	71 (65–87)	0.39
CVP, mmHg	8 (5–12)	7 (6–11)	9 (7–13)	0.59
Resuscitation before HSI				
Fluids, mL	3360 (2525–3394)	3552 (3164–3786)	3290 (2518–3576)	0.71
Noradrenaline dose, mcg/kg/min	0.11 (0.08–0.17)	0.1 (0.06–0.14)	0.15 (0.1–0.27)	0.003
Duration of noradrenaline infusion, h	14 (5–23)	14 (7–23)	11 (3–21)	0.23
Dobutamine, *n* (%)	9 (10%)	6 (9%)	3 (12%)	0.85
Dobutamine dose, mcg/kg/min	9.2 (7.6–12.1)	8.9 (7.8–12.1)	9.5 (7.6–13.0)	0.68
Acid-base parameters				
pH (arterial)	7.36 (7.28–7.47)	7.39 (7.31–7.46)	7.31 (7.21–7.37)	0.007
PaCO2, mmHg	31 (27–36)	30 (27–35)	33 (29–37)	0.18
PaO2, mmHg	93 (72–126)	88 (69–120)	107 (77–127)	0.44
PaO2/FiO2, mmHg	250 (173–332)	263 (195–374)	191 (145–254)	0.04
Perfusion parameters				
Lactate, mmol/L	2.3 (1.2–4.1)	2.6 (1.9–4.2)	3.7 (2.0–5.4)	0.3
Mottling score	0 (0;4)	0 (0;3)	0 (0;4)	< 0.001
0	62 (69%)	51 (80%)	11 (40%)	n/a
1	10 (11%)	8 (13%)	2 (8%)	n/a
2	14 (16%)	4 (6%)	10 (40%)	n/a
3	3 (3%)	1 (2%)	2 (8%)	n/a
4	1 (1%)	0	1 (4%)	n/a

Values are expressed as median (interquartile range) or count (percentage). Mottling score is reported as median (range)

*APACHE II* Acute Physiology, Age, Chronic Health Evaluation II; *SOFA* Sequential Organ Failure Assessment; *n/a* not applicable

The majority of patients were over 60 years old, 60% were male and 35% had an abdominal source of sepsis. At the time of imaging, 69% of patients had no visible mottling around the knee area.

### Skin microcirculatory indices and mottling

**Fig. 2:**
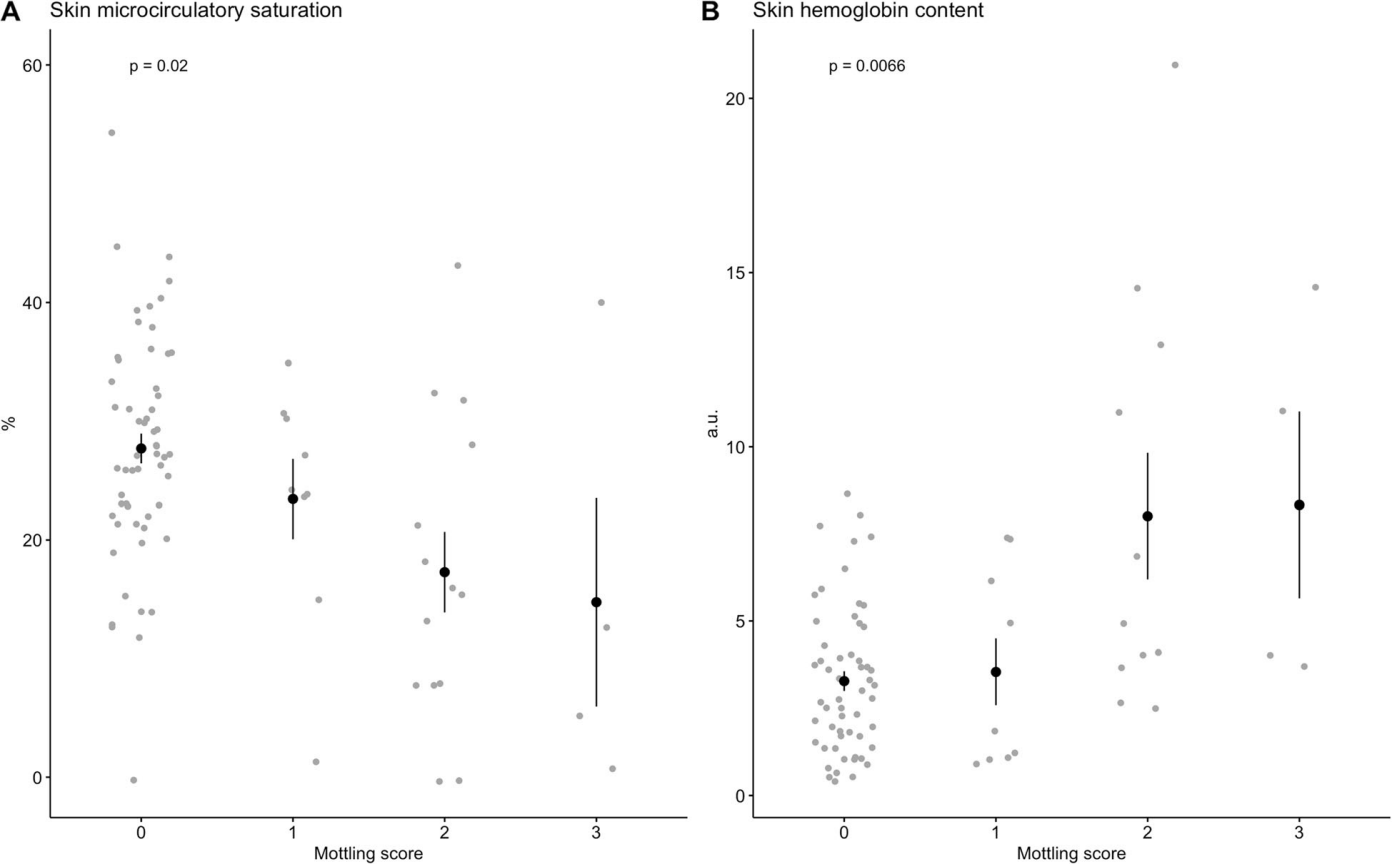
Skin microcirculatory indices obtained from hyperspectral imaging according to mottling score. **a** Skin microcirculatory oxygen saturation, expressed as percentage, and **b** skin total hemoglobin concentration, expressed in arbitrary units (a.u.), according to the mottling score. Data are presented as the means ± standard error

Patients with a MS of 0, 1, 2, and 3 had μHbSO_2_ of 27 (22–33)%, 24 (15–30)%, 15 (8–21)% and 5 (1–13)%, respectively. The Kruskal-Wallis test showed that μHbSO_2_ was significantly different in patients depending on the MS (*p* = 0.02; Fig. 2a). Skin μHbtot in the imaged area was 2.89 (1.65–4.43), 1.84 (1.08–6.15), 4.92 (3.84–11.95), and 7.52 (3.94–11.92) a.u. in patients with a MS of 0, 1, 2, and 3, respectively (*p* = 0.007; Fig. 2b). Further, pairwise comparisons revealed that μHbSO_2_ was significantly lower and μHbtot was significantly higher in patients with an MS of 2 compared to those with an MS of 0 (*p* = 0.02 for both comparisons). No correlation was found between the blood hemoglobin concentration and μHbtot or the partial pressure of oxygen in the arterial blood and μHbSO_2._

### Biomarkers of endothelial activation and skin microcirculatory indices

**Fig. 3:**
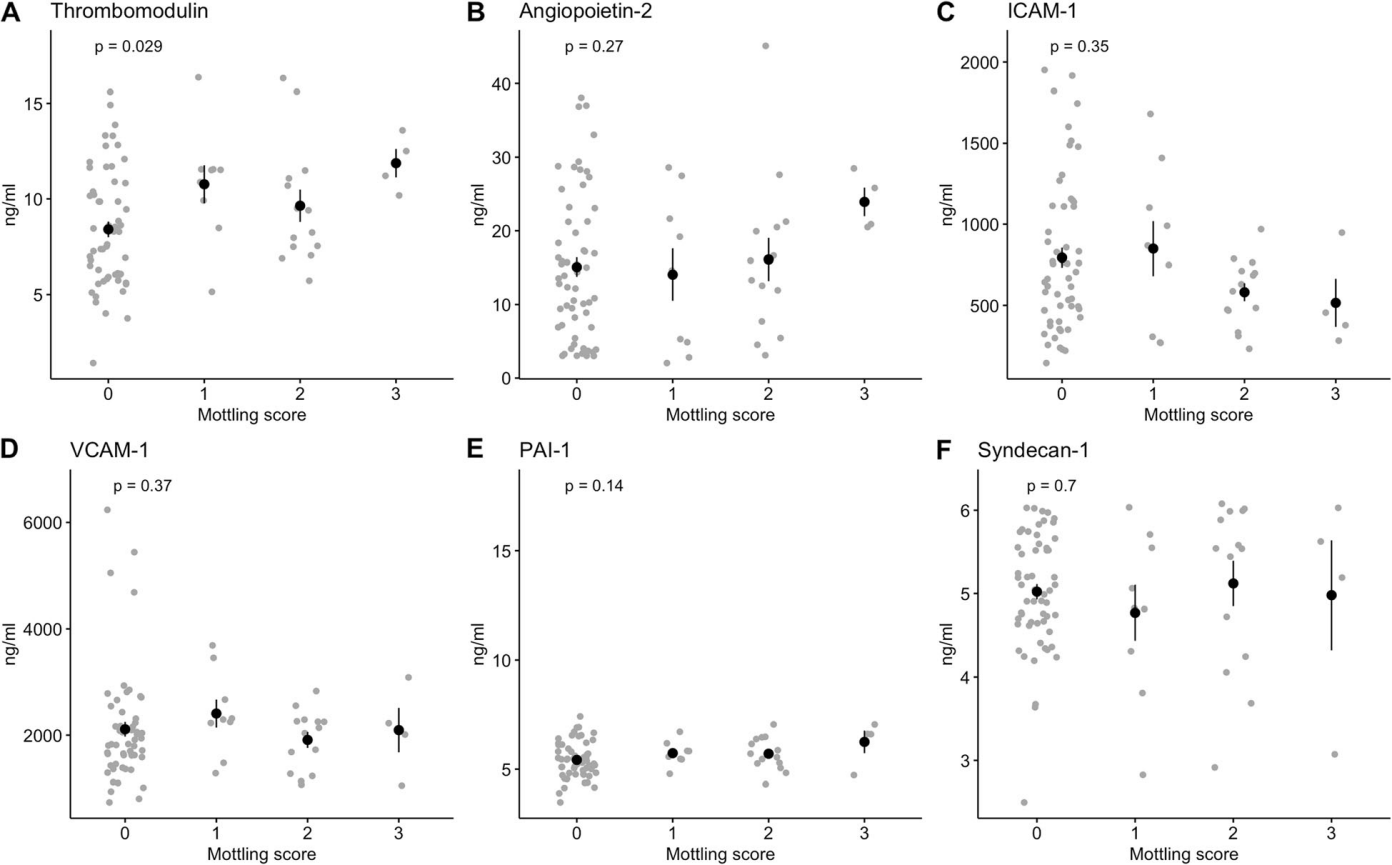
**a**–**f** Relationship between mottling score and biomarkers of endothelial activation. Data are presented as means ± standard error

**Fig. 4:**
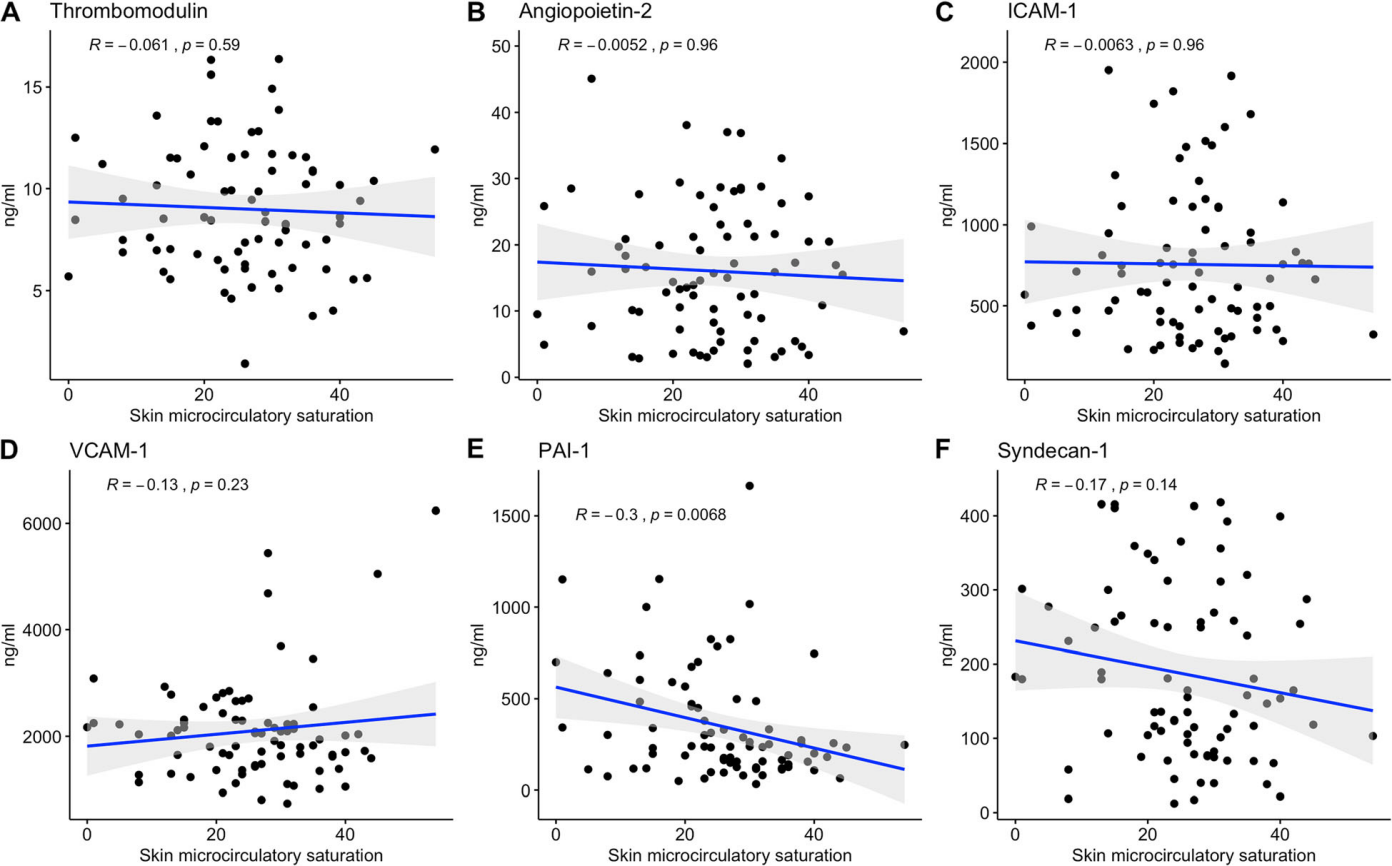
**a**–**f** Relationship between skin microcirculatory saturation and biomarkers of endothelial activation. Data are presented as the means ± standard error

We tested the association between biomarkers representing various aspects of endothelial function, MS, and μHbSO_2_. Plasma concentrations of thrombomodulin were significantly higher in patients with an MS of 1 and 3 compared to patients with no mottling (*p* = 0.03). None of the other biomarkers showed a significant association with the extent of mottling (Fig. 3). Figure 4 describes the relationship between the six biomarkers and μHbSO_2_. We found a negative correlation of μHbSO_2_ with PAI-1 levels (rho = − 0.3; *p* = 0.007).

### Skin microcirculatory indices and outcome

Skin μHbSO_2_ was higher in patients who survived the episode of septic shock than in those who died (28 (23–33)% vs 16 (10.5–21)%; *p* = 0.0003). In the imaged skin area, μHbtot was similar between the two groups [survivors, 2.89 (1.64–4.95) a.u.; non-survivors, 4.02 (3.14–46.73) a.u.; *p* = 0.07]. In the univariate analysis shown in Table 2, both MS and μHbSO_2_ were predictive of 28-day mortality with *C* statistics of 0.76 and 0.79, respectively. A mottling score of 2 or more was predictive of death with a sensitivity of 80% and a specificity of 65%, whereas a μHbSO_2_ cutoff of 26% was 84% sensitive and 78% specific. Table 3 shows the relationship between the probability of 28-day survival, mottling score, skin microcirculatory indices, and biomarkers of endothelial activation modeled using multivariate logistic regression. As the likelihood of survival in sepsis is influenced by disease severity, analysis of the odds of survival was adjusted for the severity of sepsis measured by SOFA score. The 28-day survival was independently associated with higher μHbSO_2_ (odds ratio 1.13, *p* = 0.007) and lower SOFA score values (odds ratio 0.64, *p* = 0.03). Although not significant, there was a trend towards lower survival in patients with an MS of 2 or more (odds ratio 0.16, *p* = 0.11). No association was detected between biomarkers of endothelial activation and 28-day survival in this model.

**Table 2:** Results of univariate analysis of the association between mottling score, skin microcirculatory oxygenation, and 28-day mortality

Risk factor	Odds ratio	95% CI	*p* value
Mottling score (0 as reference)			< 0.001
1	1.75	0.23–8.96	0.53
2	15.31	4.12–68.11	< 0.001
3	18.37	2.07–398.52	0.02
Skin microcirculatory saturation	0.90	0.85–0.95	< 0.001

*CI* confidence interval

**Table 3:** Multivariate logistic regression model for an association between mottling score, skin microcirculatory indices, and 28-day mortality

Variable	Odds ratio	95% CI	*p* value
Mottling score (0 as reference)			
1	1.77	0.22–19.30	0.61
2	0.16	0.02–1.42	0.11
3	0.07	0–6.42	0.29
Skin microcirculatory saturation	1.12	1.04–1.23	0.008
Skin hemoglobin content	0.84	0.62–1.07	0.20
Thrombomodulin	0.89	0.65–1.20	0.46
Angiopoietin-2	1.07	0.99–1.16	0.10
ICAM-1	1.00	1.0	0.89
VCAM-1	1.00	1.0	0.24
PAI-1	1.00	1.0	0.48
Syndecan-1	1.00	0.99–1.01	0.98
SOFA score	0.64	0.41–0.92	0.03

*CI* confidence interval, *ICAM-1* intracellular adhesion molecule-1, *VCAM-1* vascular cell adhesion molecule-1, *PAI-1* plasminogen activator inhibitor-1, *SOFA* Sequential Organ Failure Assessment

## Discussion

Our study found that in patients with septic shock, higher mottling scores were associated with increased skin total hemoglobin content and decreased oxygen saturation. However, a significant overlap of the values of skin microcirculatory indices was found between different MS categories, especially between patients with MS of 0 and 1. Similar to HSI-based oxygenation measurements in this study, progressive mottling has been associated with lower knee area tissue saturation values by Ait-Outfella and colleagues [4]. Although the mottling score has been proposed as a 6-point scale by the same group, when comparing MS and tissue oxygenation, these authors pooled the grades 0–1, 2–3, and 4–5 for analysis. Our study confirms that broader scoring categories when evaluating the extent of mottling are justified, as there is an overlap.

The association between poor peripheral perfusion and outcome has been reported in many studies [9, 14, 15]. In our cohort of patients with septic shock, a mottling score of 2 or 3 and a decrease in μHbSO_2_ were strong predictors of 28-day mortality. Mottling extending over the patella or above was a sensitive but not specific predictor of poor outcome with a coefficient of discrimination of 0.76. This finding confirms the results obtained by Dumas and colleagues who reported a similar discrimination coefficient in the validation cohort of their study [16]. The advantage of direct quantitative evaluation of skin oxygenation over MS as a prognostic factor is its greater specificity (78% vs 65%). Even in patients with no visible mottling (MS 0), the median value of μHbSO_2_ was 27%, which was only 1% higher than the cutoff value for increased mortality. This finding shows that some patients with significant microcirculatory hypoxia cannot be identified by visual assessment. On the other hand, the risk of death did not differ among patients with mottling scores of 0 and 1, which again indicates the redundancy of an MS of 1. The prognostic value of low tissue oxygenation measured early in the course of critical illness is consistent with previous research, where hemodynamically resuscitated septic patients with persisting low thenar muscle oxygen saturation measured by near-infrared spectroscopy were found to have worse survival [17, 18].

The lack of difference between skin hemoglobin content between survivors and non-survivors was unexpected. Intravital microscopy shows decreased functional capillary density in septic patients [19]. Due to the loss of functional capillaries, more oxygen extraction occurs in normal flowing capillaries, resulting in low hemoglobin oxygen saturation, which could explain the areas of low μHbSO_2_ but no change in skin hemoglobin content. However, this explanation remains speculative, as we have no data regarding flow rates in the capillaries in our cohort of patients and cannot draw conclusions about oxygen transport.

Decreased capillary perfusion in sepsis is thought to be caused by endothelial activation, and multiple previous studies have found a positive association between circulating biomarkers of endothelial activation and risk of death and organ dysfunction [20, 21]. There is less information regarding the link between levels of circulating biomarkers and underlying pathophysiologic mechanisms of organ damage. In our study, we examined the association between levels of biomarkers of endothelial activation and skin microcirculatory oxygenation. The examined biomarkers were chosen to represent hemostatic (thrombomodulin, PAI-1) and vascular integrity (angiopoietin-2, VCAM-1, ICAM-1), as well as the shedding of the glycocalyx (syndecan-1) [22]. Only PAI-1 levels, which reflect decreased fibrinolysis and possible fibrin deposit formation, were negatively related to skin microcirculatory hemoglobin oxygen saturation. Although previous studies have reported increased PAI-1 levels in septic patients with unfavorable outcomes and overt disseminated intravascular coagulation [23], the finding of high PAI-1 levels in the context of peripheral hypoperfusion has not been described previously. Patients with higher mottling scores also had higher levels of thrombomodulin, a natural anticoagulant, but this finding was less reliable as there was no clear dose-response relationship between the extent of mottling and circulating thrombomodulin levels. Both of these findings point to a hypocoagulable state with increased fibrinolysis in patients with extensive mottling and low μHbSO_2_. The degree of peripheral tissue dysoxia was not related to the changes in circulating biomarkers of vascular integrity and shedding of the glycocalyx in our cohort. No evidence was found that any of the investigated biomarkers increased the predictive value of μHbSO_2_ on mortality.

In our study, all patients were recruited from a single intensive care unit which makes the results difficult to generalize to other populations. Variation in skin chromophores, such as melanin and bilirubin, can influence the precision of HSI-based measurements. Therefore, studies confirming our results in diverse intensive care populations are needed. Based on previous research, we expected to observe the whole range of mottling scores in our 89-patient cohort. However, the recruited cohort had only 4 patients with an MS of 3 and a single patient with an MS of 4. As a result, we were only able to investigate the associations of skin microcirculatory indices, endothelial biomarkers, and mottling scores of 0 to 3, and our results apply only to this patient group. Another limitation of our dataset is that cardiac output variables were not available as were measured only in a minority of patients. Although this limitation makes the description of the patient cohort less complete, other studies investigating mottling in septic patients have not found significant differences in cardiac index and central venous oxygen saturation in patients with and without mottling [3, 5].

This study shows that HSI-based methodology can be used at the bedside for the assessment of blood pooling and microcirculatory hemoglobin oxygen saturation in the skin of patients admitted to intensive care units. HSI allows the mapping of spatial variation of hemoglobin concentration and saturation and improves the understanding of physiology and prognosis of the patient. However, we only measured μHbSO_2_ at a single time point early in the course of sepsis and have no data to support HSI-based measurements of skin oxygenation as a dynamic guide for the assessment of microcirculatory derangements. The important next step would be to investigate the longitudinal change in relation to the progression of organ dysfunction during intensive care unit stay.

## Conclusions

Higher mottling scores are associated with lower μHbSO_2_, but there is a significant overlap between the grades of mottling. μHbSO_2_ is a quantitative measure of peripheral oxygenation and is more specific than MS in predicting 28-day mortality. High circulating levels of PAI-1, a marker of endothelial damage, have been shown to be related to low μHbSO_2_.

## Additional file

Additional file 1: Hyperspectral image acquisition and analysis. (DOCX 295 kb)

## Data Availability

All the data supporting our findings are available from the corresponding author upon reasonable request.

## Abbreviations

MS: Mottling score
PAI-1: Tissue plasminogen activator inhibitor-1
ICAM-1: Soluble intercellular adhesion molecule-1
VCAM-1: Soluble vascular cell adhesion molecule-1
OR: Odds ratio
CI: Confidence interval
HSI: Hyperspectral imaging
μHbSO2: Microcirculatory hemoglobin oxygen saturation
APACHE II: Acute Physiology and Chronic Health Evaluation II
SOFA: Sequential Organ Failure Assessment
μHbtot: Relative total hemoglobin concentration
a.u.: Arbitrary units
