# Supplementary material for: Relationship of mottling score, skin microcirculatory perfusion indices and biomarkers of endothelial dysfunction in patients with septic shock: an observational study
Source: Crit Care. 2019 Sep 11;23:311. doi: 10.1186/s13054-019-2589-0 (PMC6739999; doi:10.1186/s13054-019-2589-0)
Supplement: Supplementary file 1 — Hyperspectral image acquisition and analysis. (DOCX 295 kb) [file 13054_2019_2589_MOESM1_ESM.docx]

**Additional file**

**Measurement of skin microcirculatory oxygen saturation using hyperspectral imaging to predict outcome in patients with septic shock: an observational study**

Sigita Kazune^1,2^*, Anastasija Caica^2,3^, Karina Volceka^2,3^, Olegs Suba^4^, Uldis Rubins^2^, Andris Grabovskis^2^

^1^ Department of Anesthesiology, Hospital of Traumatology and Orthopedics, 22 Duntes Street, Riga LV-1013, LATVIA

^2^ Laboratory of Biophotonics, Institute of Atomic Physics and Spectroscopy, University of Latvia, 3 Jelgavas Street, Riga, LV-1004, LATVIA

^3^ Department of Human and Animal Physiology, Faculty of Biology, University of Latvia, 1 Jelgavas Street, Riga, LV-1004, LATVIA

^4^ Clinic of Toxicology and Sepsis, Riga East University Hospital, 2 Hipokrata Street, Riga, LV-1038, LATVIA

**Hyperspectral image acquisition and analysis**

For the acquisition of hyperspectral images (HSI) we used a tunable solid-state liquid crystal filter camera Nuance EX (PerkinElmer, USA). The camera was equipped with a 60 mm f2.8D Micro Nikkor lens (Nikon, Japan) adjusted for a field of view of 10x10 cm to capture snapshots at a constant distance of 30 cm. For illumination, the camera was assembled with a coaxial ring of 5x185 lumen halogen tungsten lamps Aluline (Philips, Netherlands) and 10x140 lumen 567.5 nm (lime color) LUXEON light-emitting diodes (Lumileds, Netherlands) that particularly amplify the optical signal in the range of hemoglobin absorption (520-580nm). To reduce glare from the stratum corneum linear polarizer film was placed in front of the light source oriented orthogonally to the camera filter’s built-in polarizer. The equipment setup is shown in Figure S1.


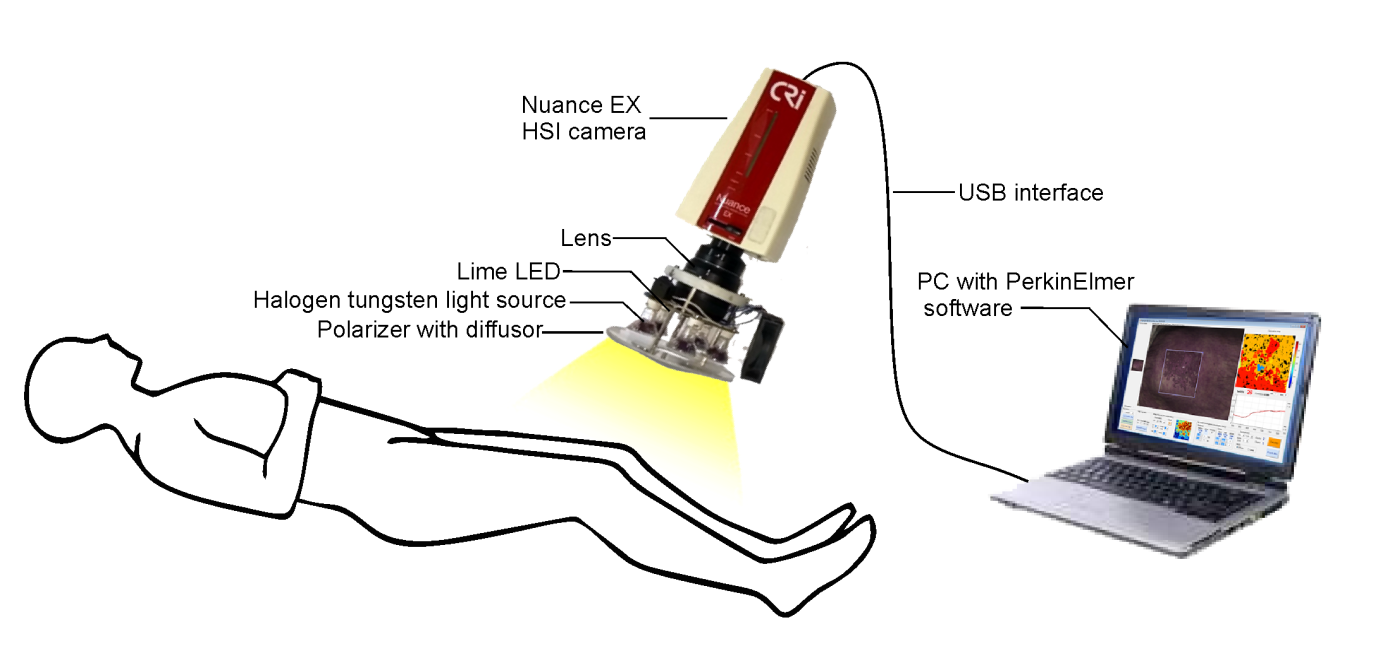


**Figure S1** The setup of equipment for hyperspectral imaging

The field of view was selected from the realtime 550 nm image on the PC, picking a region of skin with most pronounced mottling. HSI were captured in the bright-field mode utilizing the 4x4 pixel binning for shorter exposure time. Before acquisition of each image, the camera software auto-exposure function was used to set the exact exposure timing for each band from the image of spectrally neutral 18% GreyCard (Kodak, USA) placed directly over the patient knee, thereby leveling the white balance and brightness of the HSI image cube otherwise affected by the changing surrounding light conditions. Each patient’s HSI data-cube contained 75 12-bit 1392x1024 pixel monochromatic images captured in a range of 450-820 nm with a step of 5 nm. All HSI cubes were saved as a set of lossless monochrome .tiff files.

Data processing

The processing of the HSI data was performed offline in semi-automatic mode using custom MATLAB (MathWorks, Natick, MA, USA) code comprising several major HSI processing stages:

- image stabilization, panning, rotation, and stretching;
- k-means clustering of the 75-dimension HSI image cube, dividing the cube into three clusters by the pixel spectral properties that correspond to the total hemoglobin content thus obtaining precise skin regions with the highest pooling of blood;
- estimating the reflectance spectra in each image pixel, by dividing the skin to grey-card images and calculating the oxygen saturation value by using the diffusion light transport model that includes parameters for three-layer skin structure and oxyhemoglobin, deoxyhemoglobin and melanin chromophore content.

We have previously validated an algorithm for calculation of microcirculatory hemoglobin oxygen saturation (μHbSO_2_) using HSI in healthy volunteers (1) and shown the feasibility of using HSI at the bedside in intensive care patients (2).

References

1. Marcinkevics Z, Rubins U, Grabovskis A, Cimurs J, Caica A. Hyperspectral evaluation of skin blood oxygen saturation at baseline and during arterial occlusion. In: Biophotonics: Photonic Solutions for Better Health Care VI. International Society for Optics and Photonics; 2018 [cited 2019 Apr 27]. p. 106851A.

2. Saknite I, Grabovskis A, Kazune S, Rubins U, Marcinkevics Z, Volceka K, et al. Novel hybrid technology for early diagnostics of sepsis. In: Multimodal Biomedical Imaging XII. 2017. p. 100570F. (Society of Photo-Optical Instrumentation Engineers (SPIE) Conference Series; vol. 10057).
